# Supplementary material for: Modeling glioblastoma heterogeneity as a dynamic network of cell states
Source: Mol Syst Biol. 2021 Sep 16;17(9):e10105. doi: 10.15252/msb.202010105 (PMC8444284; doi:10.15252/msb.202010105)
Supplement: Supplementary file 6 — Source Data for Figure 5 [file MSB-17-e10105-s004.zip › Figure5A_sourcedata/GSEA_3071/nosigdb_stateV.GseaPreranked.1622795159954/VANGRONINGEN_MES.html]

Details for gene set VANGRONINGEN\_MES[GSEA]

|  || Dataset | state43071 |
| Phenotype | NoPhenotypeAvailable |
| Upregulated in class | na\_neg |
| GeneSet | VANGRONINGEN\_MES |
| Enrichment Score (ES) | -0.504627 |
| Normalized Enrichment Score (NES) | -3.3615973 |
| Nominal p-value | 0.0 |
| FDR q-value | 0.0 |
| FWER p-Value | 0.0 |
Table: GSEA Results Summary

  

Fig 1: Enrichment plot: VANGRONINGEN\_MES      
 Profile of the Running ES Score & Positions of GeneSet Members on the Rank Ordered List

  

| PROBE | GENE SYMBOL | GENE\_TITLE | RANK IN GENE LIST | RANK METRIC SCORE | RUNNING ES | CORE ENRICHMENT || 1 | NFIA |  |  | 42 | 1.604 | -0.0014 | No |
| 2 | PTN |  |  | 70 | 1.482 | -0.0004 | No |
| 3 | SCRG1 |  |  | 87 | 1.404 | 0.0025 | No |
| 4 | DLX2 |  |  | 104 | 1.357 | 0.0052 | No |
| 5 | DLX1 |  |  | 128 | 1.314 | 0.0063 | No |
| 6 | GAS1 |  |  | 154 | 1.263 | 0.0068 | No |
| 7 | NFIC |  |  | 172 | 1.227 | 0.0087 | No |
| 8 | TMEFF2 |  |  | 224 | 1.142 | 0.0035 | No |
| 9 | PTGFRN |  |  | 232 | 1.134 | 0.0070 | No |
| 10 | SEL1L3 |  |  | 242 | 1.128 | 0.0101 | No |
| 11 | TNC |  |  | 246 | 1.123 | 0.0144 | No |
| 12 | PROM1 |  |  | 356 | 1.008 | -0.0030 | No |
| 13 | SPRY1 |  |  | 389 | 0.959 | -0.0052 | No |
| 14 | DUSP6 |  |  | 408 | 0.944 | -0.0047 | No |
| 15 | LITAF |  |  | 412 | 0.939 | -0.0012 | No |
| 16 | IQGAP2 |  |  | 520 | 0.851 | -0.0189 | No |
| 17 | A2M |  |  | 532 | 0.846 | -0.0175 | No |
| 18 | APP |  |  | 570 | 0.818 | -0.0213 | No |
| 19 | GPC6 |  |  | 577 | 0.814 | -0.0190 | No |
| 20 | PTPRG |  |  | 693 | 0.750 | -0.0387 | No |
| 21 | JAM3 |  |  | 704 | 0.745 | -0.0375 | No |
| 22 | TRAM2 |  |  | 719 | 0.739 | -0.0370 | No |
| 23 | KDELC2 |  |  | 723 | 0.738 | -0.0344 | No |
| 24 | SSBP4 |  |  | 735 | 0.729 | -0.0335 | No |
| 25 | FAT1 |  |  | 740 | 0.727 | -0.0311 | No |
| 26 | SHROOM3 |  |  | 747 | 0.724 | -0.0292 | No |
| 27 | ROBO1 |  |  | 771 | 0.713 | -0.0307 | No |
| 28 | MAML2 |  |  | 784 | 0.708 | -0.0300 | No |
| 29 | ATP1B1 |  |  | 824 | 0.689 | -0.0348 | No |
| 30 | FGFR1 |  |  | 862 | 0.673 | -0.0393 | No |
| 31 | SOX9 |  |  | 905 | 0.658 | -0.0448 | No |
| 32 | PRCP |  |  | 983 | 0.626 | -0.0575 | No |
| 33 | FBN2 |  |  | 1029 | 0.614 | -0.0638 | No |
| 34 | PHTF2 |  |  | 1094 | 0.594 | -0.0740 | No |
| 35 | TRIL |  |  | 1127 | 0.586 | -0.0779 | No |
| 36 | EPHA3 |  |  | 1150 | 0.578 | -0.0798 | No |
| 37 | ITM2B |  |  | 1244 | 0.547 | -0.0960 | No |
| 38 | ITPR1 |  |  | 1282 | 0.537 | -0.1010 | No |
| 39 | SPRY4 |  |  | 1283 | 0.536 | -0.0987 | No |
| 40 | EFEMP2 |  |  | 1300 | 0.532 | -0.0996 | No |
| 41 | PTBP1 |  |  | 1311 | 0.530 | -0.0993 | No |
| 42 | NES |  |  | 1325 | 0.527 | -0.0996 | No |
| 43 | ELAVL1 |  |  | 1391 | 0.508 | -0.1104 | No |
| 44 | KCTD12 |  |  | 1494 | 0.481 | -0.1287 | No |
| 45 | PLEKHH2 |  |  | 1595 | 0.461 | -0.1467 | No |
| 46 | RAB31 |  |  | 1604 | 0.459 | -0.1463 | No |
| 47 | CRTAP |  |  | 1686 | 0.439 | -0.1606 | No |
| 48 | EGR1 |  |  | 1691 | 0.438 | -0.1595 | No |
| 49 | CRISPLD1 |  |  | 1697 | 0.437 | -0.1586 | No |
| 50 | PDGFC |  |  | 1759 | 0.425 | -0.1689 | No |
| 51 | WWTR1 |  |  | 1794 | 0.419 | -0.1739 | No |
| 52 | PLEKHA2 |  |  | 1805 | 0.417 | -0.1741 | No |
| 53 | PDIA4 |  |  | 1807 | 0.416 | -0.1725 | No |
| 54 | HOMER1 |  |  | 1835 | 0.413 | -0.1761 | No |
| 55 | ADGRE5 |  |  | 1842 | 0.412 | -0.1755 | No |
| 56 | HTRA1 |  |  | 1958 | 0.395 | -0.1968 | No |
| 57 | MEST |  |  | 1980 | 0.391 | -0.1993 | No |
| 58 | MANF |  |  | 2023 | 0.384 | -0.2060 | No |
| 59 | CBFB |  |  | 2035 | 0.382 | -0.2065 | No |
| 60 | GNS |  |  | 2099 | 0.374 | -0.2175 | No |
| 61 | TSC22D2 |  |  | 2132 | 0.370 | -0.2223 | No |
| 62 | ANXA1 |  |  | 2165 | 0.366 | -0.2271 | No |
| 63 | SPRED1 |  |  | 2166 | 0.366 | -0.2255 | No |
| 64 | PCOLCE2 |  |  | 2215 | 0.361 | -0.2335 | No |
| 65 | ACTA2 |  |  | 2227 | 0.359 | -0.2342 | No |
| 66 | ADAM9 |  |  | 2228 | 0.359 | -0.2326 | No |
| 67 | NID1 |  |  | 2297 | 0.350 | -0.2447 | No |
| 68 | DPY19L1 |  |  | 2347 | 0.342 | -0.2530 | No |
| 69 | MRC2 |  |  | 2378 | 0.338 | -0.2575 | No |
| 70 | ITM2C |  |  | 2558 | 0.316 | -0.2920 | No |
| 71 | FNDC3B |  |  | 2617 | 0.308 | -0.3022 | No |
| 72 | SYPL1 |  |  | 2625 | 0.307 | -0.3023 | No |
| 73 | CXCL12 |  |  | 2633 | 0.306 | -0.3023 | No |
| 74 | SASH1 |  |  | 2634 | 0.306 | -0.3010 | No |
| 75 | SRPX |  |  | 2646 | 0.305 | -0.3019 | No |
| 76 | PRRX1 |  |  | 2674 | 0.302 | -0.3060 | No |
| 77 | FZD1 |  |  | 2869 | 0.283 | -0.3435 | No |
| 78 | ELK4 |  |  | 2971 | 0.272 | -0.3625 | No |
| 79 | CALU |  |  | 3001 | 0.269 | -0.3672 | No |
| 80 | PDIA6 |  |  | 3003 | 0.269 | -0.3662 | No |
| 81 | ANTXR1 |  |  | 3037 | 0.265 | -0.3716 | No |
| 82 | SLC30A7 |  |  | 3208 | -0.252 | -0.4045 | No |
| 83 | IL6ST |  |  | 3233 | -0.257 | -0.4082 | No |
| 84 | MMP2 |  |  | 3234 | -0.257 | -0.4071 | No |
| 85 | ATP8B2 |  |  | 3243 | -0.258 | -0.4076 | No |
| 86 | ASPH |  |  | 3273 | -0.262 | -0.4122 | No |
| 87 | TRIM5 |  |  | 3328 | -0.271 | -0.4219 | No |
| 88 | STEAP1 |  |  | 3334 | -0.273 | -0.4217 | No |
| 89 | LUZP1 |  |  | 3380 | -0.278 | -0.4295 | No |
| 90 | TSC22D3 |  |  | 3396 | -0.281 | -0.4312 | No |
| 91 | SLC35F5 |  |  | 3398 | -0.281 | -0.4302 | No |
| 92 | CTDSP2 |  |  | 3410 | -0.284 | -0.4312 | No |
| 93 | SPCS3 |  |  | 3415 | -0.284 | -0.4307 | No |
| 94 | SFT2D1 |  |  | 3422 | -0.285 | -0.4307 | No |
| 95 | C6orf120 |  |  | 3448 | -0.289 | -0.4344 | No |
| 96 | PPIB |  |  | 3507 | -0.301 | -0.4447 | No |
| 97 | RGS10 |  |  | 3512 | -0.302 | -0.4442 | No |
| 98 | HLX |  |  | 3542 | -0.306 | -0.4487 | No |
| 99 | OGFRL1 |  |  | 3543 | -0.307 | -0.4474 | No |
| 100 | KLF10 |  |  | 3555 | -0.308 | -0.4482 | No |
| 101 | IFITM2 |  |  | 3585 | -0.312 | -0.4527 | No |
| 102 | GJA1 |  |  | 3602 | -0.316 | -0.4545 | No |
| 103 | ITGB1 |  |  | 3614 | -0.317 | -0.4553 | No |
| 104 | KANK2 |  |  | 3634 | -0.321 | -0.4577 | No |
| 105 | C1orf198 |  |  | 3662 | -0.325 | -0.4617 | No |
| 106 | GSN |  |  | 3695 | -0.331 | -0.4667 | No |
| 107 | GDF15 |  |  | 3713 | -0.333 | -0.4686 | No |
| 108 | HIBADH |  |  | 3742 | -0.338 | -0.4727 | No |
| 109 | CNN3 |  |  | 3751 | -0.339 | -0.4729 | No |
| 110 | TNS1 |  |  | 3761 | -0.342 | -0.4732 | No |
| 111 | SVIL |  |  | 3763 | -0.342 | -0.4719 | No |
| 112 | CTSO |  |  | 3774 | -0.344 | -0.4724 | No |
| 113 | SYNJ2 |  |  | 3787 | -0.347 | -0.4733 | No |
| 114 | SEPT10 |  |  | 3805 | -0.350 | -0.4752 | No |
| 115 | VIM |  |  | 3830 | -0.354 | -0.4784 | No |
| 116 | SSR3 |  |  | 3832 | -0.355 | -0.4771 | No |
| 117 | SDCBP |  |  | 3840 | -0.357 | -0.4769 | No |
| 118 | TMED9 |  |  | 3851 | -0.359 | -0.4774 | No |
| 119 | RAB29 |  |  | 3864 | -0.361 | -0.4782 | No |
| 120 | KLF4 |  |  | 3866 | -0.361 | -0.4768 | No |
| 121 | TUBB6 |  |  | 3904 | -0.368 | -0.4826 | No |
| 122 | TSPAN4 |  |  | 3916 | -0.369 | -0.4832 | No |
| 123 | TRAM1 |  |  | 3935 | -0.375 | -0.4852 | No |
| 124 | HIST1H2AC |  |  | 3949 | -0.377 | -0.4862 | No |
| 125 | SERPINE2 |  |  | 3951 | -0.377 | -0.4847 | No |
| 126 | JAK1 |  |  | 4019 | -0.393 | -0.4964 | No |
| 127 | ARMCX2 |  |  | 4021 | -0.393 | -0.4949 | No |
| 128 | CD59 |  |  | 4024 | -0.394 | -0.4936 | No |
| 129 | YAP1 |  |  | 4041 | -0.398 | -0.4951 | No |
| 130 | EHD2 |  |  | 4051 | -0.399 | -0.4951 | No |
| 131 | RNH1 |  |  | 4065 | -0.402 | -0.4960 | No |
| 132 | CLIC4 |  |  | 4075 | -0.404 | -0.4960 | No |
| 133 | PHLDB2 |  |  | 4076 | -0.404 | -0.4943 | No |
| 134 | TMBIM4 |  |  | 4099 | -0.410 | -0.4969 | No |
| 135 | LAMB1 |  |  | 4115 | -0.413 | -0.4981 | No |
| 136 | ATP2B1 |  |  | 4126 | -0.416 | -0.4983 | No |
| 137 | LIFR |  |  | 4158 | -0.426 | -0.5026 | No |
| 138 | GALNT10 |  |  | 4169 | -0.429 | -0.5028 | Yes |
| 139 | ELK3 |  |  | 4175 | -0.430 | -0.5019 | Yes |
| 140 | PXDN |  |  | 4176 | -0.430 | -0.5000 | Yes |
| 141 | TGFB1I1 |  |  | 4177 | -0.430 | -0.4982 | Yes |
| 142 | CREB3L2 |  |  | 4178 | -0.430 | -0.4963 | Yes |
| 143 | ITGAV |  |  | 4207 | -0.442 | -0.5000 | Yes |
| 144 | MYADM |  |  | 4217 | -0.445 | -0.4998 | Yes |
| 145 | CMTM3 |  |  | 4220 | -0.446 | -0.4983 | Yes |
| 146 | APOE |  |  | 4229 | -0.449 | -0.4979 | Yes |
| 147 | FAM129A |  |  | 4244 | -0.454 | -0.4988 | Yes |
| 148 | FSTL1 |  |  | 4262 | -0.460 | -0.5002 | Yes |
| 149 | MYL12B |  |  | 4267 | -0.462 | -0.4990 | Yes |
| 150 | RCN1 |  |  | 4276 | -0.467 | -0.4985 | Yes |
| 151 | ATP6V0E1 |  |  | 4302 | -0.473 | -0.5015 | Yes |
| 152 | LASP1 |  |  | 4310 | -0.475 | -0.5008 | Yes |
| 153 | F2R |  |  | 4316 | -0.476 | -0.4997 | Yes |
| 154 | LTBP1 |  |  | 4322 | -0.477 | -0.4987 | Yes |
| 155 | B2M |  |  | 4341 | -0.485 | -0.5002 | Yes |
| 156 | NOTCH2 |  |  | 4355 | -0.488 | -0.5006 | Yes |
| 157 | EDNRA |  |  | 4361 | -0.489 | -0.4995 | Yes |
| 158 | HNMT |  |  | 4379 | -0.494 | -0.5008 | Yes |
| 159 | POLR2L |  |  | 4382 | -0.496 | -0.4990 | Yes |
| 160 | CYR61 |  |  | 4384 | -0.497 | -0.4971 | Yes |
| 161 | KDM5B |  |  | 4392 | -0.499 | -0.4963 | Yes |
| 162 | RAP1A |  |  | 4401 | -0.501 | -0.4957 | Yes |
| 163 | TFE3 |  |  | 4415 | -0.505 | -0.4961 | Yes |
| 164 | IFITM3 |  |  | 4424 | -0.509 | -0.4955 | Yes |
| 165 | LIPA |  |  | 4436 | -0.516 | -0.4955 | Yes |
| 166 | SURF4 |  |  | 4459 | -0.524 | -0.4976 | Yes |
| 167 | RAP1B |  |  | 4465 | -0.525 | -0.4963 | Yes |
| 168 | ARL1 |  |  | 4471 | -0.525 | -0.4950 | Yes |
| 169 | L3HYPDH |  |  | 4484 | -0.532 | -0.4951 | Yes |
| 170 | TJP1 |  |  | 4489 | -0.536 | -0.4936 | Yes |
| 171 | CCDC80 |  |  | 4510 | -0.546 | -0.4952 | Yes |
| 172 | NANS |  |  | 4520 | -0.549 | -0.4946 | Yes |
| 173 | ADAMTS5 |  |  | 4528 | -0.554 | -0.4936 | Yes |
| 174 | SLC39A14 |  |  | 4530 | -0.554 | -0.4914 | Yes |
| 175 | SLC38A2 |  |  | 4536 | -0.557 | -0.4900 | Yes |
| 176 | BTN3A2 |  |  | 4546 | -0.562 | -0.4893 | Yes |
| 177 | PYGL |  |  | 4554 | -0.567 | -0.4883 | Yes |
| 178 | LMAN1 |  |  | 4569 | -0.572 | -0.4886 | Yes |
| 179 | SCPEP1 |  |  | 4589 | -0.579 | -0.4899 | Yes |
| 180 | ARL4A |  |  | 4593 | -0.582 | -0.4879 | Yes |
| 181 | WIPI1 |  |  | 4599 | -0.589 | -0.4864 | Yes |
| 182 | ANXA5 |  |  | 4616 | -0.601 | -0.4870 | Yes |
| 183 | LOXL2 |  |  | 4624 | -0.605 | -0.4857 | Yes |
| 184 | S1PR3 |  |  | 4625 | -0.605 | -0.4831 | Yes |
| 185 | NR3C1 |  |  | 4673 | -0.630 | -0.4898 | Yes |
| 186 | P4HA1 |  |  | 4689 | -0.641 | -0.4900 | Yes |
| 187 | KDELR2 |  |  | 4700 | -0.645 | -0.4892 | Yes |
| 188 | SEMA3C |  |  | 4703 | -0.646 | -0.4868 | Yes |
| 189 | MYDGF |  |  | 4704 | -0.646 | -0.4840 | Yes |
| 190 | HSPA5 |  |  | 4714 | -0.652 | -0.4829 | Yes |
| 191 | FZD2 |  |  | 4719 | -0.658 | -0.4809 | Yes |
| 192 | PLAGL1 |  |  | 4723 | -0.661 | -0.4786 | Yes |
| 193 | PLS3 |  |  | 4771 | -0.692 | -0.4850 | Yes |
| 194 | PCSK5 |  |  | 4774 | -0.694 | -0.4824 | Yes |
| 195 | ACTN1 |  |  | 4775 | -0.695 | -0.4794 | Yes |
| 196 | F2RL2 |  |  | 4778 | -0.697 | -0.4767 | Yes |
| 197 | LGALS1 |  |  | 4784 | -0.699 | -0.4747 | Yes |
| 198 | RAB13 |  |  | 4786 | -0.701 | -0.4718 | Yes |
| 199 | DUSP5 |  |  | 4796 | -0.707 | -0.4706 | Yes |
| 200 | IGFBP6 |  |  | 4800 | -0.715 | -0.4681 | Yes |
| 201 | PPIC |  |  | 4802 | -0.716 | -0.4651 | Yes |
| 202 | PCDH18 |  |  | 4803 | -0.717 | -0.4620 | Yes |
| 203 | NEK7 |  |  | 4807 | -0.719 | -0.4595 | Yes |
| 204 | ARPC1B |  |  | 4818 | -0.730 | -0.4583 | Yes |
| 205 | BNC2 |  |  | 4819 | -0.730 | -0.4552 | Yes |
| 206 | PRDX4 |  |  | 4821 | -0.732 | -0.4522 | Yes |
| 207 | PALLD |  |  | 4839 | -0.749 | -0.4523 | Yes |
| 208 | NPC2 |  |  | 4841 | -0.750 | -0.4493 | Yes |
| 209 | LRP10 |  |  | 4843 | -0.751 | -0.4462 | Yes |
| 210 | ANXA6 |  |  | 4859 | -0.762 | -0.4459 | Yes |
| 211 | RHOC |  |  | 4868 | -0.776 | -0.4441 | Yes |
| 212 | PXDC1 |  |  | 4872 | -0.780 | -0.4413 | Yes |
| 213 | DUSP14 |  |  | 4895 | -0.804 | -0.4422 | Yes |
| 214 | DSE |  |  | 4903 | -0.818 | -0.4401 | Yes |
| 215 | SOSTDC1 |  |  | 4914 | -0.825 | -0.4385 | Yes |
| 216 | AJUBA |  |  | 4916 | -0.826 | -0.4351 | Yes |
| 217 | CKAP4 |  |  | 4919 | -0.831 | -0.4319 | Yes |
| 218 | ELF1 |  |  | 4927 | -0.841 | -0.4296 | Yes |
| 219 | ZFP36L1 |  |  | 4929 | -0.843 | -0.4262 | Yes |
| 220 | KLF6 |  |  | 4933 | -0.849 | -0.4231 | Yes |
| 221 | ITPRIPL2 |  |  | 4943 | -0.860 | -0.4212 | Yes |
| 222 | TMEM263 |  |  | 4946 | -0.861 | -0.4178 | Yes |
| 223 | FLNA |  |  | 4948 | -0.862 | -0.4143 | Yes |
| 224 | FKBP14 |  |  | 4954 | -0.872 | -0.4115 | Yes |
| 225 | RIN2 |  |  | 4958 | -0.875 | -0.4083 | Yes |
| 226 | CALD1 |  |  | 4960 | -0.877 | -0.4047 | Yes |
| 227 | DDR2 |  |  | 4971 | -0.886 | -0.4028 | Yes |
| 228 | KDELR3 |  |  | 4974 | -0.893 | -0.3993 | Yes |
| 229 | FUCA2 |  |  | 4977 | -0.896 | -0.3958 | Yes |
| 230 | IGFBP5 |  |  | 4978 | -0.897 | -0.3919 | Yes |
| 231 | RRBP1 |  |  | 4981 | -0.898 | -0.3884 | Yes |
| 232 | PAPSS2 |  |  | 4983 | -0.901 | -0.3847 | Yes |
| 233 | HSPB1 |  |  | 4992 | -0.920 | -0.3823 | Yes |
| 234 | CTSB |  |  | 5007 | -0.948 | -0.3810 | Yes |
| 235 | ITGA10 |  |  | 5016 | -0.960 | -0.3784 | Yes |
| 236 | QSOX1 |  |  | 5018 | -0.968 | -0.3744 | Yes |
| 237 | OSTC |  |  | 5019 | -0.972 | -0.3702 | Yes |
| 238 | EMP1 |  |  | 5023 | -0.979 | -0.3666 | Yes |
| 239 | FLRT2 |  |  | 5029 | -0.989 | -0.3633 | Yes |
| 240 | DLC1 |  |  | 5036 | -1.013 | -0.3601 | Yes |
| 241 | ANXA2 |  |  | 5055 | -1.036 | -0.3592 | Yes |
| 242 | UGDH |  |  | 5056 | -1.037 | -0.3547 | Yes |
| 243 | SERPINH1 |  |  | 5059 | -1.050 | -0.3505 | Yes |
| 244 | CPED1 |  |  | 5078 | -1.090 | -0.3494 | Yes |
| 245 | EVA1A |  |  | 5081 | -1.098 | -0.3450 | Yes |
| 246 | UAP1 |  |  | 5082 | -1.100 | -0.3402 | Yes |
| 247 | CAPN2 |  |  | 5088 | -1.120 | -0.3363 | Yes |
| 248 | SHC1 |  |  | 5094 | -1.133 | -0.3324 | Yes |
| 249 | PLXDC2 |  |  | 5096 | -1.138 | -0.3277 | Yes |
| 250 | SQSTM1 |  |  | 5099 | -1.149 | -0.3231 | Yes |
| 251 | FILIP1L |  |  | 5103 | -1.158 | -0.3186 | Yes |
| 252 | COL11A1 |  |  | 5110 | -1.178 | -0.3147 | Yes |
| 253 | COL5A2 |  |  | 5112 | -1.189 | -0.3098 | Yes |
| 254 | PDLIM1 |  |  | 5114 | -1.191 | -0.3048 | Yes |
| 255 | FAM114A1 |  |  | 5127 | -1.230 | -0.3018 | Yes |
| 256 | RBMS1 |  |  | 5129 | -1.240 | -0.2966 | Yes |
| 257 | CSRP1 |  |  | 5130 | -1.243 | -0.2912 | Yes |
| 258 | TIMP1 |  |  | 5134 | -1.251 | -0.2864 | Yes |
| 259 | ATP2B4 |  |  | 5139 | -1.270 | -0.2817 | Yes |
| 260 | OLFML2A |  |  | 5140 | -1.270 | -0.2762 | Yes |
| 261 | LMNA |  |  | 5141 | -1.277 | -0.2706 | Yes |
| 262 | PHLDA3 |  |  | 5150 | -1.320 | -0.2665 | Yes |
| 263 | STK38L |  |  | 5162 | -1.381 | -0.2627 | Yes |
| 264 | GPX8 |  |  | 5171 | -1.418 | -0.2581 | Yes |
| 265 | MICAL2 |  |  | 5176 | -1.441 | -0.2527 | Yes |
| 266 | COL12A1 |  |  | 5186 | -1.484 | -0.2480 | Yes |
| 267 | THBS1 |  |  | 5197 | -1.538 | -0.2433 | Yes |
| 268 | COL4A2 |  |  | 5198 | -1.545 | -0.2366 | Yes |
| 269 | TFPI |  |  | 5202 | -1.557 | -0.2305 | Yes |
| 270 | NQO1 |  |  | 5210 | -1.596 | -0.2249 | Yes |
| 271 | NRP1 |  |  | 5211 | -1.609 | -0.2179 | Yes |
| 272 | TPM1 |  |  | 5215 | -1.643 | -0.2114 | Yes |
| 273 | BGN |  |  | 5216 | -1.654 | -0.2042 | Yes |
| 274 | VCL |  |  | 5222 | -1.678 | -0.1979 | Yes |
| 275 | OLFML3 |  |  | 5224 | -1.700 | -0.1907 | Yes |
| 276 | TPBG |  |  | 5229 | -1.755 | -0.1839 | Yes |
| 277 | EGFR |  |  | 5230 | -1.765 | -0.1762 | Yes |
| 278 | TM4SF1 |  |  | 5235 | -1.812 | -0.1692 | Yes |
| 279 | MYL12A |  |  | 5239 | -1.826 | -0.1618 | Yes |
| 280 | SNAI2 |  |  | 5241 | -1.903 | -0.1537 | Yes |
| 281 | CDH11 |  |  | 5245 | -1.943 | -0.1459 | Yes |
| 282 | TPM2 |  |  | 5247 | -1.967 | -0.1376 | Yes |
| 283 | MGP |  |  | 5252 | -2.037 | -0.1295 | Yes |
| 284 | CFH |  |  | 5257 | -2.141 | -0.1210 | Yes |
| 285 | FBN1 |  |  | 5263 | -2.234 | -0.1123 | Yes |
| 286 | COL6A2 |  |  | 5265 | -2.276 | -0.1026 | Yes |
| 287 | COL4A1 |  |  | 5266 | -2.279 | -0.0927 | Yes |
| 288 | COL5A1 |  |  | 5274 | -2.455 | -0.0834 | Yes |
| 289 | COL6A1 |  |  | 5283 | -2.747 | -0.0731 | Yes |
| 290 | MGST1 |  |  | 5286 | -2.788 | -0.0614 | Yes |
| 291 | FN1 |  |  | 5287 | -2.866 | -0.0489 | Yes |
| 292 | COL3A1 |  |  | 5292 | -3.630 | -0.0340 | Yes |
| 293 | COL6A3 |  |  | 5293 | -3.722 | -0.0178 | Yes |
| 294 | COL1A1 |  |  | 5294 | -4.144 | 0.0002 | Yes |
Table: GSEA details [plain text format]

  

Fig 2: VANGRONINGEN\_MES: Random ES distribution      
 Gene set null distribution of ES for **VANGRONINGEN\_MES**
